# Identification of mTOR as a primary resistance factor of the IAP antagonist AT406 in hepatocellular carcinoma cells

**DOI:** 10.18632/oncotarget.14326

**Published:** 2016-12-28

**Authors:** Mao-Chuan Zhen, Fu-Qiang Wang, Shao-Feng Wu, Yi-Lin Zhao, Ping-Guo Liu, Zhen-Yu Yin

**Affiliations:** ^1^ Department of Hepatobiliary Surgery, Zhongshan Hospital of Xiamen University, Fujian Provincial Key Laboratory of Chronic Liver Disease and Hepatocellular Carcinoma, Xiamen, Fujian, 361004, China; ^2^ Department of Tumor Interventional Radiology, Zhongshan Hospital of Xiamen University, Xiamen, Fujian, 361004, China

**Keywords:** inhibitor of apoptosis (IAP) proteins, AT406, Mcl-1, mTOR, OSI-027

## Abstract

Dysregulation of inhibitor of apoptosis (IAP) proteins (IAPs) in hepatocellular carcinoma (HCC) is often associated with poor prognosis. Here we showed that AT406, an IAP antagonist, was cytotoxic and pro-apoptotic to both established (HepG2, SMMC-7721 lines) and primary HCC cells. Activation of mTOR could be a key resistance factor of AT406 in HCC cells. mTOR inhibition (by OSI-027), kinase-dead mutation or knockdown remarkably enhanced AT406-induced lethality in HCC cells. Reversely, forced-activation of mTOR by adding SC79 or exogenous expressing a constitutively active S6K1 (T389E) attenuated AT406-induced cytotoxicity against HCC cells. We showed that AT406 induced degradation of IAPs (cIAP-1 and XIAP), but didn't affect another anti-apoptosis protein Mcl-1. Co-treatment of OSI-027 caused simultaneous Mcl-1 downregulation to overcome AT406's resistance. Significantly, shRNA knockdown of Mcl-1 remarkably facilitated AT406-induced apoptosis in HCC cells. *In vivo*, AT406 oral administration suppressed HepG2 tumor growth in nude mice. Its activity was potentiated with co-administration of OSI-027. We conclude that mTOR could be a key resistance factor of AT406 in HCC cells.

## INTRODUCTION

Hepatocellular carcinoma (HCC) is one of the leading cause of cancer-related mortalities in the World [1, 2]. Only few early-stage and locally-defined HCCs could be possibly cured via surgery resection [1, 2]. The prognosis of HCC patients with recurrent and/or metastatic tumors is extremely poor [1, 2]. Molecularly-targeted therapy has become the research focus of the HCC therapy [1, 2]. Groups all over the world are exploring novel and more efficient anti-HCC agents [1, 2].

Apoptosis evasion is a major characteristic of cancer cells [3]. Inhibitor of apoptosis (IAP) proteins (IAPs), including the X-linked IAP (XIAP) and cellular IAP-1 and 2 (cIAP-1/2), participate in cancer cell progression [4]. XIAP directly inhibits several caspases, including caspase-3 and -7, and -9 [4, 5]. cIAP-1/2 are capable of disrupting the pro-apoptotic protein signalling assemble [4–6]. Existing studies have reported mutations, amplifications and chromosomal translocations of *IAP* genes in HCCs [7], which are often associated with patients’ poor prognosis [5, 7]. Therefore, IAPs represent attractive therapeutic targets for HCC [8].

Recently, a novel and orally bio-available small molecular IAP antagonist, AT406, was developed [9]. AT406 binds directly to several key IAPs to block their activities [9]. Preclinical cancer studies have shown that AT406 could provoke cancer cell apoptosis by blocking IAPs, activating caspases, and inhibiting NFκB signalings [10, 11]. It is being tested in Phase I clinical trial of its safety, pharmacokinetics, and pharmacodynamics in human [12].

Another aim of this study is to identify possible AT406's key resistance factors. mTOR (mammalian target of rapamycin) signaling is often dysregulated and hyper-activated in HCC [13], which plays pivotal roles in cancer initiation, progression and chemo-resistance [14, 15]. mTOR lies in two multiple protein complexes: the mTOR complex 1 (mTORC1, rapamycin-sensitive) and mTOR complex 2 (mTORC2) [14, 15]. Both are important for cancer cell survival and apoptosis-resistance [14, 15]. Here we show that mTOR could be a key resistance factor of AT406. mTOR inhibition, on the other hand, dramatically sensitizes HCC cells to the IAP antagonist.

## RESULTS

### The mTOR kinase inhibitor OSI-027 potentiates AT406's cytotoxicity in HCC cells

**Figure 1:**
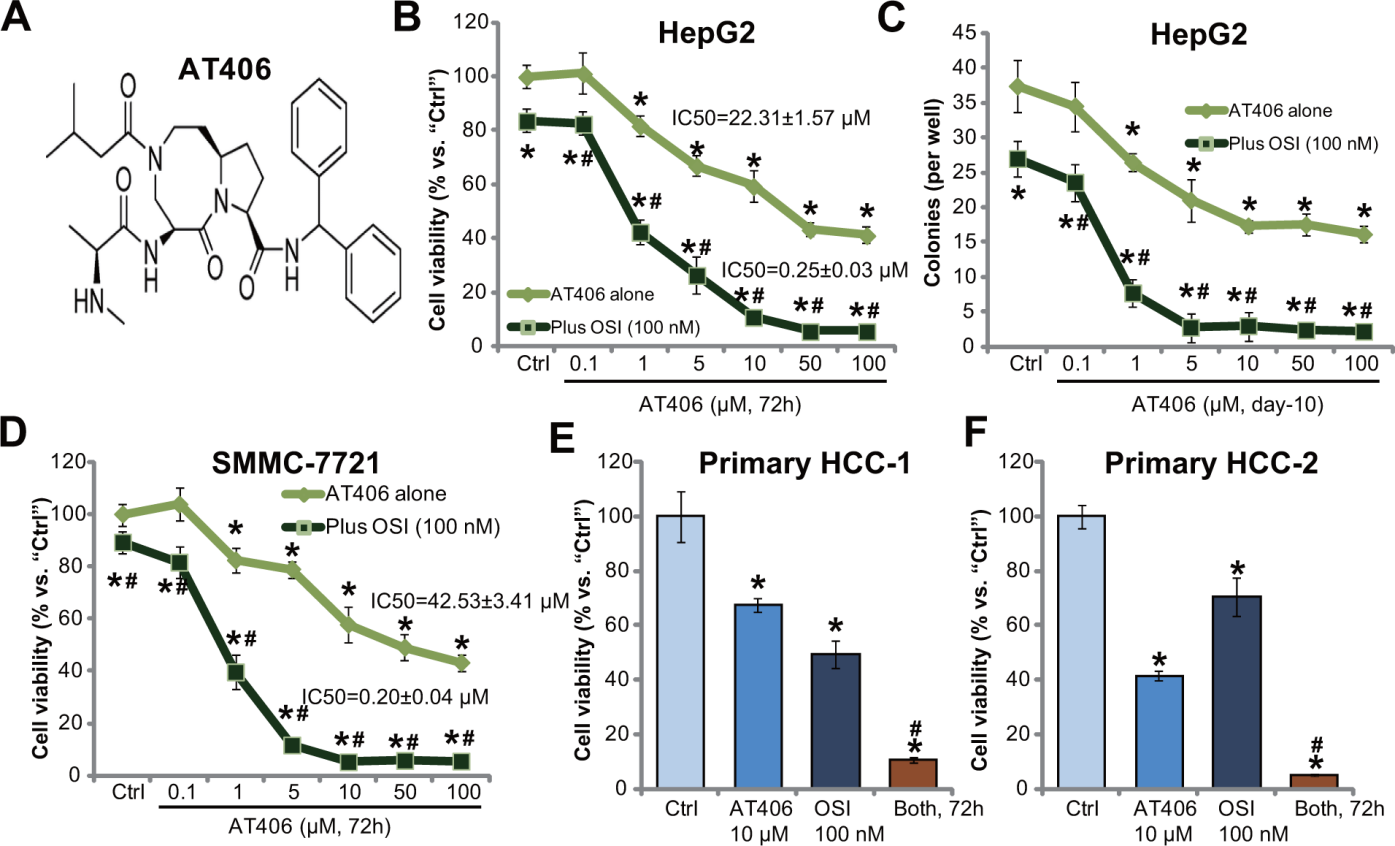
OSI-027 potentiates AT406's cytotoxicity in HCC cells Established HCC cell lines (HepG2 and SMMC-7721) or the primary human HCC cells (two lines, “HCC-1/-2”) were treated with AT406 (see molecular structure in (**A**) at applied concentrations) or plus OSI-027 (“OSI”, 100 nM), cells were further cultured and subjected to MTT assay (**B**, **D**–**F**) or clonogenic assay (**C**, for HepG2 cells) to evaluate cell survival. Data were means of three independent experiments ± SD (Same for all figures). IC-50 was calculated by the GraphPad Prism software using a sigmoidal dose-response curve model. CalcuSyn software was utilized to calculate Combination Index (CI). “Ctrl” indicated untreated control group (Same for all figures). *indicated statistically significant differences as compared to “Ctrl” group. ^#^indicated statistically significant differences as compared to “AT406” only group.

Figure 1A demonstrates the molecular structure of AT406, which has also been shown in other studies [10, 11, 16]. HepG2 cells were treated with applied concentrations of AT406, and MTT assay results in Figure 1B demonstrated that AT406 inhibited HepG2 cell survival in a dose-dependent manner. Meanwhile, the number of viable HepG2 colonies was decreased by AT406 (1 and 10 μM) (Figure 1C). The AT406-induced cytotoxicity against HepG2 cells was, however, relatively moderate (Figure 1B and 1C). The IC-50 was 22.31 ± 1.57 μM (Figure 1B and 1C). Intriguingly, co-treatment with OSI-027, a mTOR kinase inhibitor [17], dramatically potentiated AT406's cytotoxicity, resulting in substantial HepG2 cell death (MTT viability reduction, Figure 1B and 1C). AT406's IC-50 decreased to 0.25 ± 0.03 μM in the presence of OSI-027 (Figure 1B). OSI-027 by itself only exerted minor cytotoxicity to HepG2 cells, with the IC-50 over 100 μM (Figure 1B and 1C, and Supplementary Figure 1). Notably, CalcuSyn software was applied to calculate Combination Index (CI) using the Chou-Talalay method [18]. The CI values for dose-response data in Figure 1B–1C were all less than 1, indicating synergism between OSI-027 and AT406 in inhibiting HepG2 cells.

In SMMC-7721 HCC cells, OSI-027 (100 nM) again dramatically facilitated AT406-induced viability reduction (Figure 1D). AT406's IC-50 in SMMC-7721 cells was 42.53 ± 3.41 μM, yet went down to 0.20 ± 0.04 μM when combined with OSI-027 (Figure 1D). The CI value was also < 1, indicating significant synergism between the two. We also tested the activity of AT406, or plus OSI-027, in the primary cancer cells. MTT assay results showed that AT406 (10 μM) and OSI-027 (100 nM) co-treatment induced dramatic viability reduction in primary human HCC cells (Line-1/-2, Figure 1E and 1F). The combination was significantly more potent than each single agent in provoking HCC cell death (Figure 1E–1F). These results demonstrate that AT406 is cytotoxic to HCC cells, and its activity could be further potentiated with co-treatment of the mTOR kinase inhibitor OSI-027.

### OSI-027 potentiates AT406-induced HCC cell apoptosis

**Figure 2:**
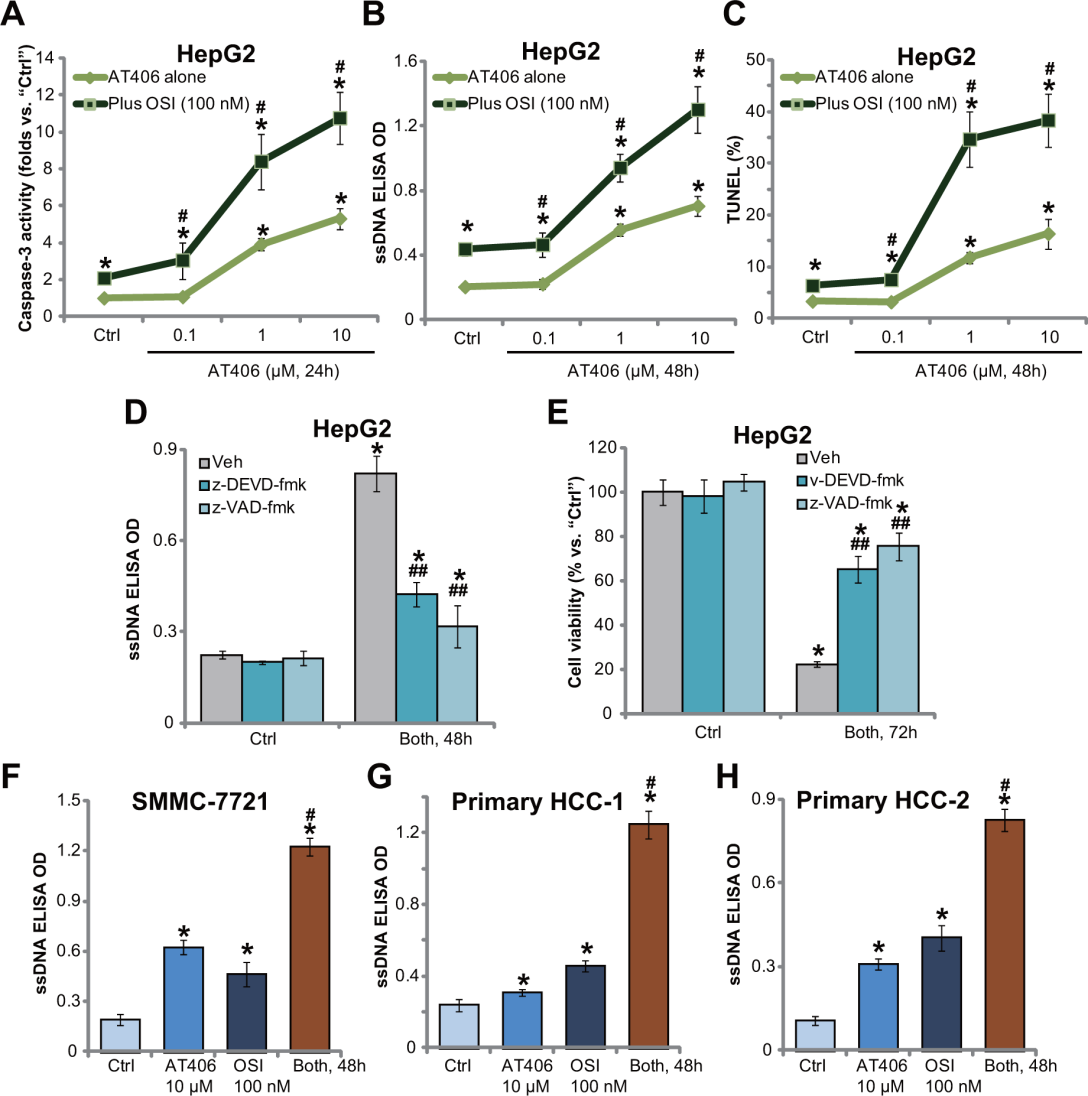
OSI-027 potentiates AT406-induced HCC cell apoptosis HCC cell lines (HepG2 and SMMC-7721) or the primary human HCC cells (“HCC-1/-2”) were treated with AT406 (at applied concentrations) or plus OSI-027 (“OSI”, 100 nM), cells were further cultured for indicated periods of time, the caspase-3 activity (**A**, for HepG2 cells) was tested; Cell apoptosis was tested by the ssDNA ELISA assay (**B**, **F**–**H**) or the TUNEL staining assay (**C**, for HepG2 cells). HepG2 cells were pretreated with the caspase-3 specific inhibitor z-DEVD-fmk (50 μM) or the general caspase inhibitor z-VAD-fmk (50 μM) for 1 hour, followed by AT406 (10 μM) plus OSI-027 (100 nM) combination treatment (“Both”), cells were further cultured and subjected to ssDNA ELISA assay of apoptosis (**D**) and MTT assay of cell viability (**E**). “Veh” stands for 0.2% of DMSO (D and E). *indicated statistically significant differences as compared to “Ctrl” group. ^#^indicated statistically significant differences as compared to “AT406” only group (A–C, F–H). ^##^indicated statistically significant differences as compared to “Both” group (D and E).

The potential effect of AT406 on HCC cancer cell apoptosis was then tested. Results in Figure 2A showed that AT406 dose-dependently increased caspase-3 activity in HepG2 cells, which was further augmented with co-treatment of OSI-027. Meanwhile, AT406 provoked apoptosis activation in HepG2 cells, which was evidenced by the ssDNA ELISA OD (Figure 2B) and TUNEL percentage increase (Figure 2C). AT406-induced apoptosis was further augmented with OSI-027 co-treatment in HepG2 cells. Further studies showed that the caspase-3 specific inhibitor z-DEVD-fmk or the general caspase inhibitor z-VAD-fmk largely attenuated combination-induced apoptosis activation (Figure 2D) and viability reduction (Figure 2E) in HepG2 cells. These results suggested that OSI-027 facilitated AT406-induced caspase-dependent apoptosis to promote HepG2 cell death. Notably, in SMMC-7721 (Figure 2F) and primary human HCC cells (Line-1/-2, Figure 2G and 2H), OSI-027 similarly potentiated AT406-induced apoptosis activation.

### mTOR knockdown or mutation potentiates AT406's cytotoxicity in HCC cells

**Figure 3:**
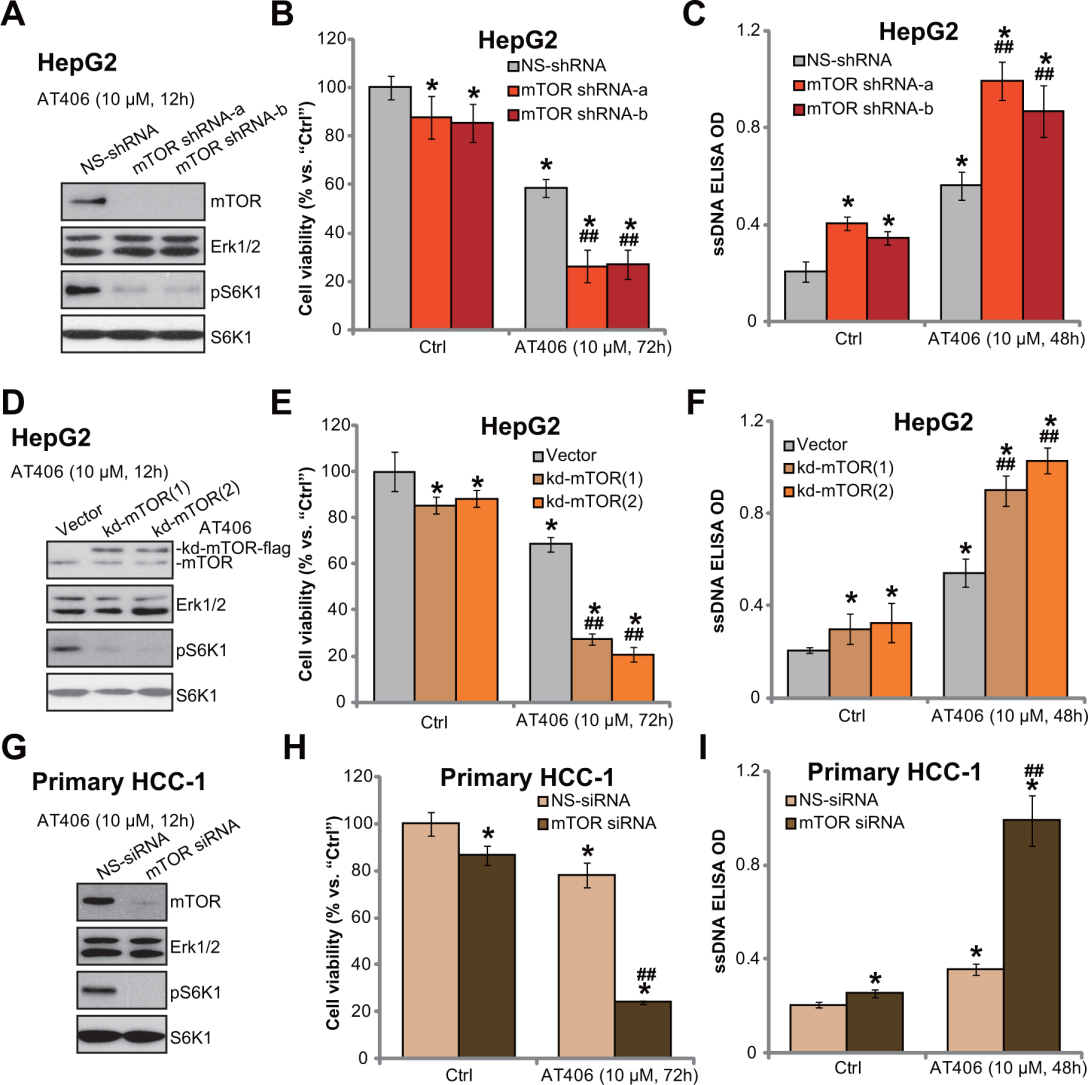
mTOR knockdown or mutation potentiates AT406's cytotoxicity in HCC cells The stable HepG2 cells, expressing mTOR shRNAs (“-a/-b”) or scramble non-sense control shRNA (“NS-shRNA”) (**A**–**C**), as well as kinase-dead (Asp-2338-Ala) mTOR (“kd-mTOR”, two lines, “-1/-2”) or the empty vector (pSuper-puro) (**D**–**F**), were stimulated with AT406 (10 μM) for indicated periods of time, expressions of listed proteins were shown (A and D); Cell viability (MTT assay, B and E) and cell apoptosis (ssDNA ELISA assay, C and F) were also tested. The primary HCC cells (“HCC-1”) transfected with scramble non-sense control siRNA (“NS-siRNA”) or mTOR siRNA (200 nM each, 24 hours) were stimulated with AT406 (10 μM) for indicated periods of time, expressions of listed proteins were shown (**G**); Cell viability (**H**) and cell apoptosis (**I**) were also tested. *indicated statistically significant differences as compared to “Ctrl” group. ^##^indicated statistically significant differences as compared to “NS-shRNA” (B and C)/“Vector” (E and F) /“NS-siRNA” group (H and I).

The above results demonstrate that mTOR inhibition by OSI-027 potentiates AT406-induced cytotoxicity in HCC cells. To exclude the possible off-target effect of OSI-027, genetic strategies were then used. First, targeted shRNAs were applied to knockdown mTOR in HepG2 cells (See methods). Western blot assay results in Figure 3A demonstrated that the two non-overlapping mTOR shRNAs (“-a/-b”) (from Dr. Liu's group [19]) dramatically downregulated mTOR in HepG2 cells. pS6K1, the indicator of mTOR activation, was almost blocked in the mTOR-silenced cells (Figure 3A). Consequently, AT406-induced cytotoxicity (Figure 3B) and apoptosis (Figure 3C) were potentiated in mTOR-silenced HepG2 cells.

Further, a kinase-dead (“kd”) mutation (Asp-2338-Ala) of mTOR (from Dr. Liu's group [19]) was introduced into HepG2 cells, and two stable HepG2 cell lines (“-1/-2”) with the mutated mTOR were established (Figure 3D). Expectably, kd-mutation of mTOR almost blocked pS6K1 in the stable cells (Figure 3D). Importantly, HepG2 cells with mTOR kd-mutation were hyper-sensitive to AT406 (more cell death and apoptosis, Figure 3E and 3F). In the primary HCC cells, targeted-siRNA were utilized to transiently knockdown mTOR. As shown in Figure 3G, mTOR expression and S6K1 phosphorylation were decreased in primary HCC cells with the mTOR siRNA. AT406-induced cytotoxicity (Figure 3H) and apoptosis (Figure 3I) were again potentiated with mTOR knockdown in the primary cancer cells. Collectively, we show that mTOR knockdown or mutation potentiates AT406's cytotoxicity in HCC cells.

### Force mTOR activation decreases AT406's sensitivity in HCC cells

**Figure 4:**
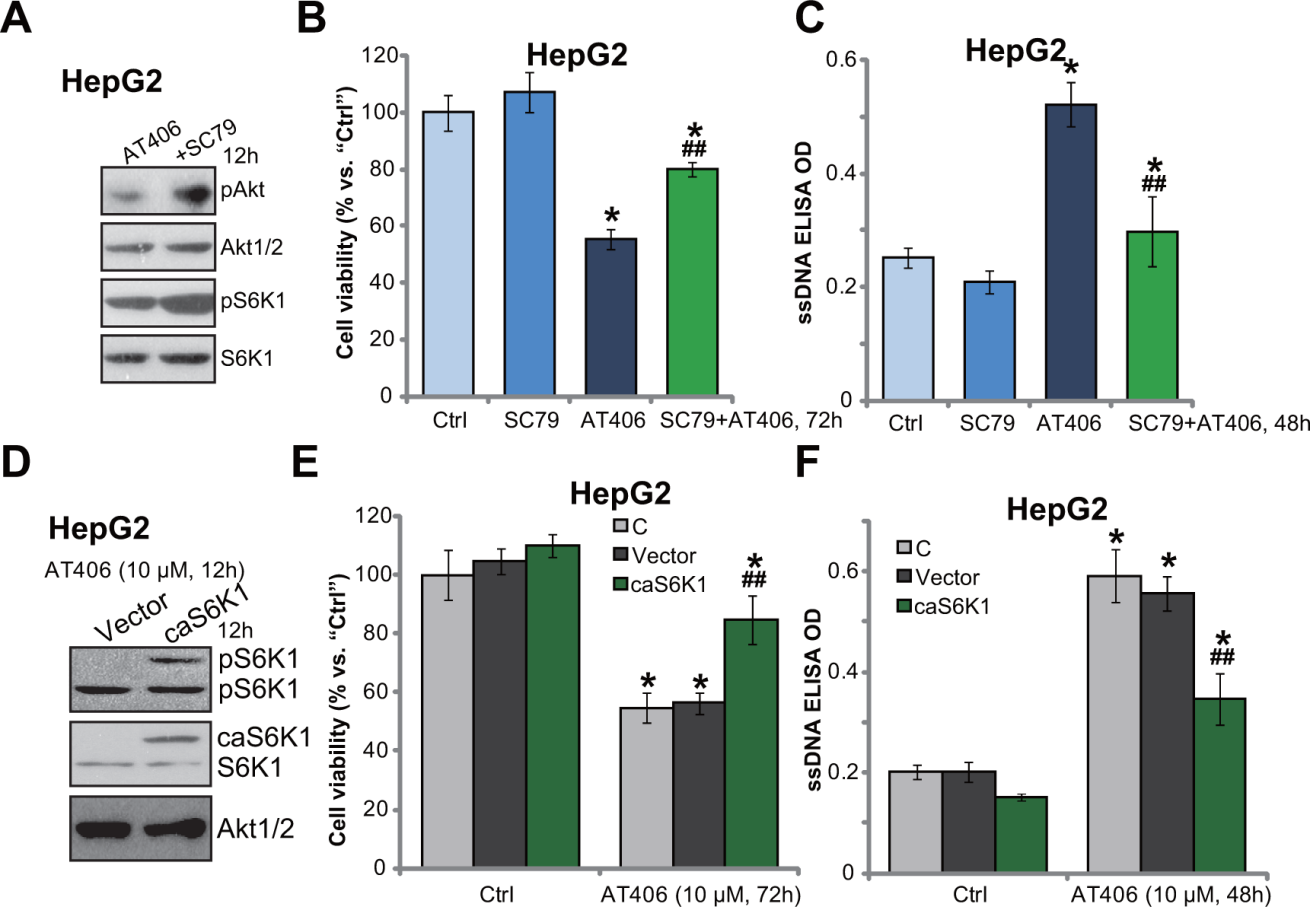
Force mTOR activation decreases AT406's sensitivity in HCC cells HepG2 cells were treated with AT406 (10 μM) and/or SC79 (10 μM) for indicated periods of time, expressions of listed proteins were tested by Western blotting assay (**A**); Cell viability (MTT assay, (**B**) and cell apoptosis (ssDNA ELISA assay, (**C**) were also tested. The stable HepG2 cells, expressing a constitutively-active S6K1 (T389E, “ca-S6K1-flag-puro”) or the empty vector, were stimulated with AT406 (10 μM) for indicated periods of time, expressions of listed proteins were shown (**D**); Cell viability (**E**) and cell apoptosis (**F**) were also tested. “C” stands for non-infected control cells (E and F). *indicated statistically significant differences as compared to “Ctrl” group. ^##^indicated statistically significant differences as compared to AT406 (10 μM) only group (B and C). ^##^indicated statistically significant differences as compared to Vector group (E and F).

Thus, mTOR inhibition (by OSI-027), kinase-dead mutation or shRNA/siRNA knockdown chemo-sensitized AT406 in HCC cells. mTOR over-activation may then decrease AT406's sensitivity. SC79, an Akt specific inhibitor [20], was utilized. Western blotting assay results in Figure 4A showed that SC79 enhanced phosphorylation of Akt (the mTORC2 activation indicator) and S6K1 (the mTORC1 activation indicator) in AT406-treated HepG2 cells. As a result, AT406-induced viability reduction (Figure 4B) and apoptosis (Figure 4C) were attenuated. We next introduced a constitutively-active S6K1 (T389E, ca-S6K1-flag-puro) [21] to HepG2 cells, and stable cells were established (Figure 4D). Expectably, S6K1 was over-activated in the stable cells, suggesting mTOR hyper-activation (Figure 4D). Consequently, AT406-indued cytotoxicity (Figure 4E) and apoptosis (Figure 4F) were largely attenuated in ca-S6K1-expressing HepG2 cells. Therefore, mTOR forced-activation decreased AT406's sensitivity in HCC cells.

### OSI-027 downregulates Mcl-1 to overcome AT406's resistance

**Figure 5:**
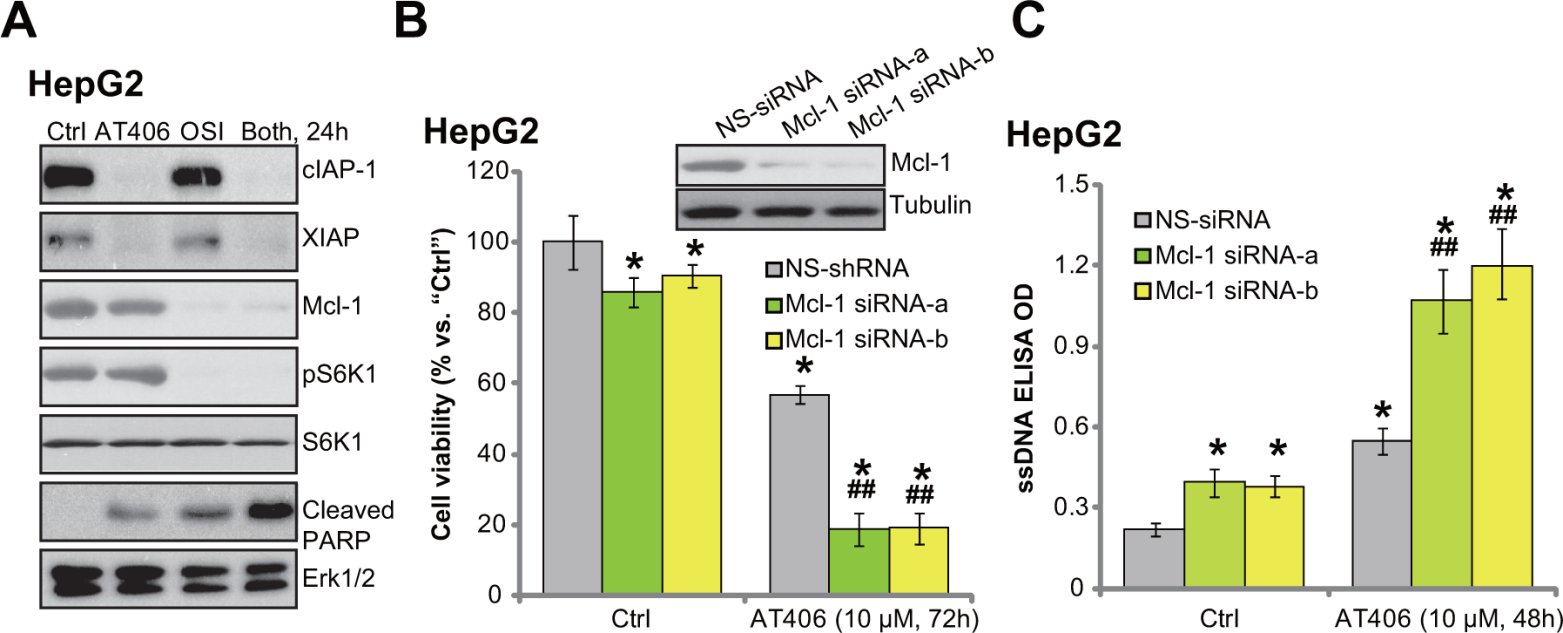
OSI-027 downregulates Mcl-1 to overcome AT406's resistance HepG2 cells were treated with AT406 (10 μM) or plus OSI-027 (“OSI”, 100 nM), cells were further cultured for indicated periods of time, expression of listed proteins was tested by Western blotting assay (**A**). HepG2 cells transfected with scramble non-sense control siRNA (“NS-siRNA”) or Mcl-1 siRNA (“-1/-2”, 200 nM each, 24 hours) were stimulated with AT406 (10 μM) for indicated periods of time, Mcl-1 expression (**B**, upper panel); Cell viability (B, lower panel) and cell apoptosis (**C**) were tested. *indicated statistically significant differences as compared to “Ctrl” group. ^##^indicated statistically significant differences as compared to “NS-siRNA” group.

We next tested the possible mechanism of OSI-027-induced AT406 sensitization. A very recent study by Shen *et al*., [22] showed that Akt-mTOR inhibition by perifosine downregulated Mcl-1 (another anti-apoptosis protein [23, 24]), which then sensitized Bcl-2 antagonist ABT-737-induced HCC cytotoxicity. Here we showed that treatment HepG2 cells with AT406 induced degradation of IAPs (cIAP-1 and XIAP) but didn't affect Mcl-1 expression (Figure 5A). Intriguingly, co-treatment with OSI-027 simantanuously induced Mcl-1 degradation (Figure 5A). To test the function of Mcl-1 in AT406's activity, we utilized siRNAs to knockdown Mcl-1 (See [22]). As demonstrated, the two non-overlapping siRNAs potently downregulated Mcl-1 in HepG2 cells (Figure 5B, upper panel). Remarkably, AT406-induced cytotoxicity (Figure 5B, lower panel) and apoptosis (Figure 5C) were dramatically augmented in Mcl-1-silenced cells. Based on these results, we proposed that although AT406 sequestered IAPs, it didn't not affect Mcl-1. Co-treatment with OSI-027 induced Mcl-1 degradation, which then overcame AT406's resistance. Indeed, only HepG2 cells treated with AT406 and OSI-027 showed a profound PARP cleavage (Figure 5A), indicating apoptosis activation (also see Figure 2). OSI-027 alone had no effect on IAP protein expression, yet it downregulated Mcl-1 in HepG2 cells (Figure 5A).

### The *in vivo* anti-tumor activity by AT406 or plus OSI-027

**Figure 6:**
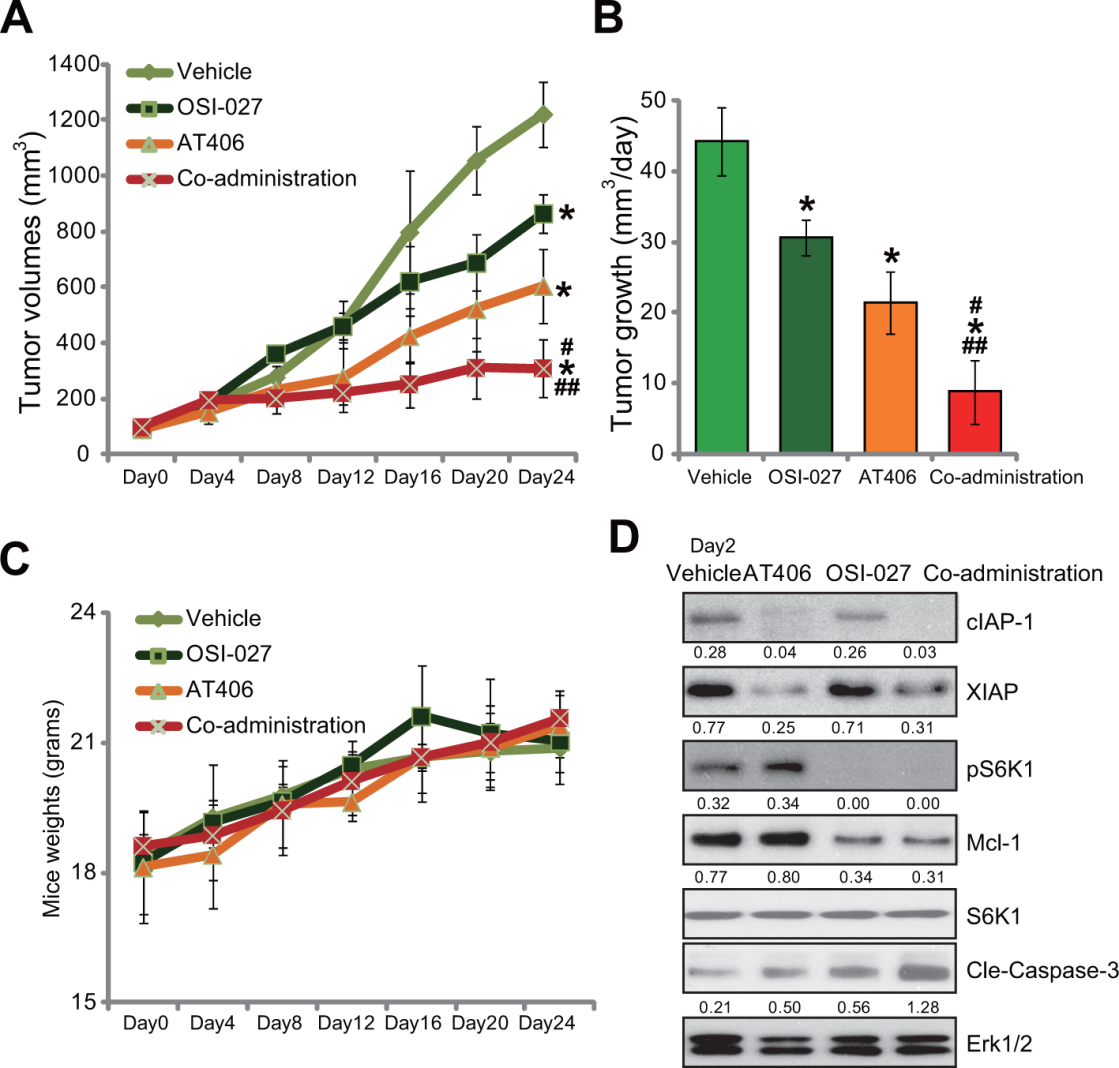
The *in vivo* anti-tumor activity by AT406 or plus OSI-027 Female nude mice bearing HepG2 tumors were treated with AT406 (50 mg/kg body weight, oral gavage, once every two days) and/or OSI-027 (10 mg/kg body weight, oral gavage, once every two days) for a total of 16 days (*n* = 10 per group), tumor volumes (in mm^3^, **A**) and mice body weights (in grams, **C**) were recorded every 4 days for a total of 24 days. Estimated daily tumor growth (in mm^3^ per day, **B**) was also presented. Two days after initial treatment, HepG2 tumors were isolated (one tumor per group), expressions of listed proteins in tumor tissues were tested by Western blotting assay (**D**). Relative expressions of cIAP-1, XIAP, Cle-Caspase-3, and Mcl-1 were quantified (vs. Erk1/2, D). S6K1 phosphorylation (vs. regular S6K1) was also quantified (D).*indicated statistically significant differences as compared to “Vehicle” group. ^#^indicated statistically significant differences as compared to “AT406” only group. ^##^indicated statistically significant differences as compared to “OSI-027” only group.

At last, the *in vivo* anti-tumor activity of AT406, or plus OSI-027, was tested. HepG2 cells were inoculated into the nude mice. Within 2–3 weeks, the xenografted tumors were established. Mice were then treated with AT406 and/or OSI-027. Results in Figure 6A showed that oral gavage of AT406 (50 mg/kg body weight, once every 2 days) inhibited growth of HepG2 tumors in nude mice. The tumor volumes in AT406-treated mice were lower than that of vehicle (“Saline”) control mice (Figure 6A). The estimated daily tumor growth (mm^3^ per day) was also decreased following AT406 administration (Figure 6B). Significantly, co-administration of OSI-027 dramatically enhanced AT406's activity *in vivo*, leading to profound HepG2 tumor growth inhibition (Figure 6A and 6B). OSI-027 alone only induced minor inhibition of HepG2 tumors (Figure 6A and 6B). Results in Figure 6C showed that mice body weight, the indicator of general health, was not significantly different between each group. Further, no apparent toxicities were noticed in the mice with AT406 and/or OSI-027 administration. These results suggested that these nude mice were well-tolerated with the tested regimens here. Western blot assay results in Figure 6D showed that only tumor tissues with the co-administration showed IAPs (cIAP-1/XIAP) degradation, Mcl-1 downregulation as well as S6K1 blockage and profound caspase-3 cleavage/activation (the indicator of cell apoptosis). Either single treatment only exerted partial or moderate activity to these signaling proteins (Figure 6D).

## DISCUSSION

The results of the current preclinical study suggest that mTOR could be a primary resistance factor of AT406 in HCC cells. mTOR inhibition (by OSI-027), kinase-dead mutation or knockdown (by siRNA or shRNA) remarkably potentiated AT406-induced cytotoxicity and apoptosis in HCC cells. On the other hand, forced activation of mTOR by SC79 or via ca-S6K1 expression attenuated the AT406's cytotoxicity in HCC cells. Importantly, AT406 oral administration only moderated suppressed HepG2 tumor growth in nude mice, and its activity *in vivo* was significantly sensitized by co-administration of OSI-027. Therefore, we propose that mTOR possibly antagonizes AT406-induced anti-HCC activity, and blockage of mTOR then sensitizes HCC cells to AT406.

Mcl-1 is a well-established anti-apoptotic Bcl-2 family protein [25, 26]. Like other Bcl-2 family members, Mcl-1 localizes to the mitochondria, where it interacts with and inhibits pro-apoptotic Bcl-2 family proteins [25, 26]. Therefore, its main function is to inhibit apoptosis activation by a number of stimuli [25, 26]. Intriguingly, unlike other Bcl-2 family members, Mcl-1 could be rapidly transcribed via PI3K-Akt-mTOR pathway [25, 26].

In the current study, we showed that OSI-027, the mTOR kinase inhibitor, induced Mcl-1 downregulation, which then sensitized AT406's cytotoxicity in HCC cells. Meanwhile, shRNA knockdown or kinase-dead mutation of mTOR also induced Mcl-1 downregulation (Data not shown) and AT406 sensitization (Figure 3) in HepG2 cells. Thus, mTOR is important for Mcl-1 expression and AT406 resistance in HCC cells. Our results are consistent with recent findings showing mTOR regulation of Mcl-1 [27, 28]. For instance, Mills et al., showed that mTORC1 dictates Mcl-1's translation [28]. Further, Koo et al., demonstrated that mTORC2, the other mTOR complex, is important for Mcl-1 stabilization [27]. The detailed underlying mechanisms of mTOR regulation of Mcl-1 expression in HCC cells may warrant further investigations.

Molecularly-targeted therapy has drawn broad attentions for better HCC treatment [1, 29]. Here we showed that combination AT406 with OSI-027 induced profound HCC cell death and apoptosis. The combined activity was superior than each single agent. *In vivo*, the two co-administration remarkably suppressed HepG2 tumor growth in nude mice. These preclinical results suggest that AT406 plus OSI-027 (or possible other mTOR kinase inhibitors [30]) could be further evaluated as potential anti-HCC agents.

## MATERIALS AND METHODS

### Chemicals and reagents

OSI-027 and AT406 were provide by Selleck Co. (Shanghai, China). Caspase inhibitors including z-VAD-fmk and z-DEVD-fmk were purchased from Sigma (Shanghai, China). All kinase antibodies were purchased from Cell Signaling Tech (Denver MA). The other antibodies utilized in this study were obtained from Santa Cruz Biotech (Santa Cruz, CA).

### Culture of human HCC cell lines

Established human HCC cell lines, including HepG2 and SMMC-7721, were cultured in RMPI-1640 medium plus 10% heat-inactive FBS with necessary antibiotics [31].

### Culture of primary human HCC cells

Two surgery removed early-stage HCC tumors (labeled as “HCC-1/-2”) were obtained from the inform-consent HCC patients (All male, 47 and 55 years old), hospitalized at authors’ institutions. The HCC tissues were washed and mechanically dissociated [31], which were then subjected to digestion via incubation in triple enzyme medium (1 × collagenase, 1 × hyaluronidase, and 1 × DNase) at room temperature for 1 hour. The primary cells were then filtered through a 70-μm nylon cell strainer (Becton Dickinson, Shanghai, China) and suspended in complete medium for primary cells [31]. The protocols requiring clinical samples were approved by the Ethics Review Board (ERB) of all authors institutions, and were in line with the principles expressed in the Declaration of Helsinki.

### MTT assay of cell viability

After treatment of cells, the cell survival was assayed by the routine MTT method as described [31–33]. The OD value of treated group was always normalized as percentage of untreated control group.

### Clonogenic assay

HepG2 cells were seeded at a density of 4000 cells/well. After overnight attachment, cells were treated with applied agents. Colony formation was determined after 10 days, and the number of remaining survival colonies was manually counted.

### ssDNA ELISA assay of apoptosis

Single strand DNA (ssDNA) in apoptotic cells was tested via a nucleosomal monoclonal antibody in an ELISA format. Briefly, cells (1 × 10^4^/well) were seeded onto 96-well plates. After treatment of cells, ssDNA content was analyzed by the ssDNA ELISA kit from Chemicon International (Temecula, CA). The ELISA OD value was recorded as a quantitative measurement of cell apoptosis.

### Caspase-3 activity assay

Following applied AT406 and/or OSI-027 treatment, caspase-Glo reagent (100 μL/well) was added. Caspase-3 activity was determined via the caspase-3 Glo kit (Promega, Shanghai, China). Caspase-3 activity in the treatment group was normalized to the fold change of the untreated control group.

### TUNEL staining assay of apoptosis

Following the applied treatment, TUNEL (Terminal deoxynucleotidyl transferase dUTP nick end labeling) *In Situ* Cell Death Detection Kit (Roche, Shanghai, China) was utilized to evaluate cell apoptosis. Cell apoptosis ratio was calculated by the TUNEL percentage (TUNEL/DAPI × 100%).

### Western blotting assay

Aliquots of 30 μg lysates per cell or tissue sample were electrophoresed on 10–12% SDS-PAGE gels, and protein lysates were then transferred to PVDF (polyvinylidene fluoride) membranes. The blots were subjected to blocking (10% BSA), followed by incubation in the designated primary antibodies and appropriate secondary antibodies. Antigen-antibody binding was detected via the enhanced chemiluminescence (ECL) reagents.

### mTOR shRNA knockdown

Two non-overlapping lentiviral mTOR shRNAs (“mTOR shRNA-a/-b”) were gifts from Dr. Liu’ [19], which were utilized to infect HepG2 cells for 24 hours. Afterwards, puromycin (5.0 μg/ml, Sigma) was added to select resistant stable HCC cells [19]. mTOR expression in the stable cells was detected by Western blotting assay.

### mTOR kinase-dead mutation

The construct with kinase-dead mTOR (“kd-mTOR-flag”, Asp-2338-Ala) and the empty vector (pSuper-puro) were gifts also from Dr. Liu's group [19]. The construct was transiently transfected into HepG2 cells via the Lipofectamine 2000 reagents (Invitrogen, Shanghai, China). Cells were then selected by puromycin (5.0 μg/mL, Sigma) for a total of 10–12 days until resistant single colony can be identified. kd-mTOR expression in stable cells was confirmed by Western blotting assay of mTOR and pS6K1.

### The constitutively active S6K1 construct and transfection

The constitutively active S6K1 (T389E, “ca-S6K1-flag-puro”) and the empty vector (pGCL-flag-puro) were gifts from Dr. Chen's group [21]. The construct was transfected to HepG2 cells via Lipofectamine 2000 protocol. Cells were also selected by puromycin (5.0 μg/mL, Sigma). “ca-S6K1” expression in the stable cells was confirmed by Western blotting assay.

### siRNA transit knockdown

The two non-overlapping siRNAs against human *Mcl-1* (Mcl-1 siRNA-1 and Mcl-1 siRNA-2) were provided by Dr. Zhao [22]. The mTOR siRNA and scramble non-sense control siRNA were purchased from Cell Signaling Tech (Shanghai, China). siRNA (200 nM each) transfection was performed via the Lipofectamine 2000 reagents. The efficiency of siRNA was determined by Western blotting assay testing expression of target protein (Mcl-1 or mTOR).

### Tumor xenograft assay

The animal protocols were approved by the Institutional Animal Care and Use Committee (IACUC) and the Ethics Review Board (ERB) of all authors institutions. The female nude mice (18–20 grams, Shanghai Experimental Animal Facility, Shanghai, China) were applied to establish HCC xenograft tumors. HepG2 cells (ten million cells per mouse) were subcutaneously injected at the right thigh of the nude mice, and treatment was started when the tumors reached an average volume of 100 mm^3^ (within two weeks). Mice were ear tagged and randomized into four groups with 10 mice per group: Vehicle (Saline), AT406 (50 mg/kg, oral gavage, once every two days) and/or OSI-027 (10 mg/kg, *i.p*. once every two days), for a total of 16 days. The mice were examine daily for toxicity/mortality relevant to treatment, and the tumor volume (in mm^3^) was calculated by the formula: volume = (width)^2^ × length/2. At the end of experiment, tumor xenografts were isolated and subjected to further signaling test.

### Statistical analysis

The data presented in this study were means ± standard deviation (SD). Statistical differences were analyzed by one-way ANOVA (SPSS). Values of *p* < 0.05 were considered statistically significant. IC-50 was calculated by the GraphPad Prism software using a sigmoidal dose-response curve model. CalcuSyn software was utilized to calculate Combination Index (CI), CI < 1 was considered as synergism.

## SUPPLEMENTARY MATERIALS FIGURE


