# Supplementary material for: Identification of mTOR as a primary resistance factor of the IAP antagonist AT406 in hepatocellular carcinoma cells
Source: Oncotarget. 2016 Dec 28;8(6):9466–75. doi: 10.18632/oncotarget.14326 (PMC5354745; doi:10.18632/oncotarget.14326)
Supplement: Supplementary file 1 [file oncotarget-08-9466-s001.pdf]

Identification of mTOR as a primary resistance factor of the IAP antagonist AT406 in hepatocellular carcinoma cells

Supplementary Materials

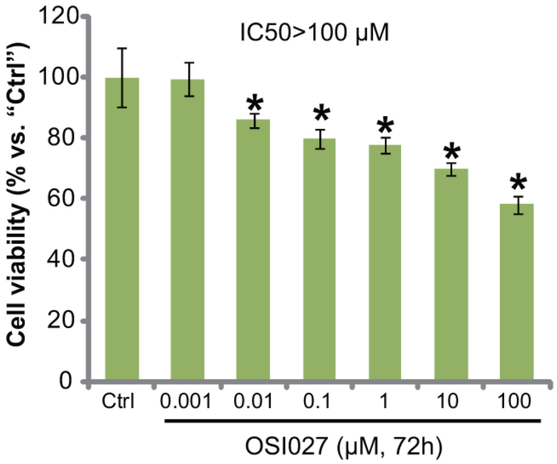

Supplementary Figure S1: HepG2 cells were treated with indicated concentration of OSI-027, cells were further cultured for 72 hours and subjected to MTT assay. Data were means of three independent experiments ± SD. "Ctrl" indicated untreated control group. *indicated statistically significant differences as compared to "Ctrl" group. IC-50 was calculated by the GraphPad Prism software using a sigmoidal dose-response curve model.
